# Spatial Correlation Network and Driving Factors of Urban Energy Eco-Efficiency from the Perspective of Human Well-Being: A Case Study of Shaanxi Province, China

**DOI:** 10.3390/ijerph20065172

**Published:** 2023-03-15

**Authors:** Meixia Wang, Qingyun Zheng, Yunxia Wang

**Affiliations:** 1 School of Economics and Management, Xi’an University of Technology, Xi’an 710054, China; 2 Business School, Shenzhen Technology University, Shenzhen 518118, China

**Keywords:** human well-being index, spatial correlation network structure, social network analysis, well-being energy eco-efficiency

## Abstract

It is very important to seek a sustainable improvement in human well-being under a limited resource supply and to promote the scientific and coordinated development of urban economic development, ecological environment protection, and human well-being. This paper constructs a human well-being index that includes economic well-being, culture and education well-being, and social development well-being as factors, and it incorporates the human well-being index into the evaluation system for urban well-being energy eco-efficiency (WEE). It uses the super-slack-based measure (SBM) model, which considers undesirable output, to measure the WEE of 10 prefecture-level cities in Shaanxi Province, China, from 2005 to 2019. The social network analysis (SNA) is used to describe the characteristics of the spatial correlation network of WEE and its spatiotemporal evolutionary trend, and the quadratic assignment procedure (QAP) analysis method is used to identify the driving factors that affect the spatial correlation network. The results show that, first, the WEE in Shaanxi is relatively low as a whole and varies greatly among regions, with the highest level in northern Shaanxi, followed by Guanzhong; the lowest level is in southern Shaanxi. Second, in Shaanxi, WEE has transcended geographical proximity into a complex, multi-threaded spatial correlation network, and Yulin is at the center of the network. Third, the network shows four sectors: the net overflow, main benefit, two-way overflow, and broker. Members in each sector have not fully exploited their advantages, and the whole network can be improved. Fourth, the differences in the economic development level, openness, industrial structure, and population are the main driving factors influencing the formation of the spatial correlation network.

## 1. Introduction

In recent years, air pollution, land degradation, shrinking ecological space, and other issues have seriously affected the health, education, and social and cultural well-being of people, especially the life satisfaction and happiness of urban residents, exacerbating the contradiction between humans and nature [1,2,3,4]. In this context, The reports of the 19th and 20th National Congresses of the Communist Party of China (CPC) clearly stated that the government should improve human well-being, improve people’s quality of life, promote green development, and promote harmonious coexistence between humans and nature. The outlines of the 14th Five-Year Plan also indicated a need to enhance human well-being and constantly realize people’s yearning for a better life. Energy eco-efficiency takes into account the environmental benefits, economic benefits, ecological benefits and social benefits of energy utilization, and it is a core indicator that reflects economic development and ecological environment conditions and for measuring the quality of regional development and the degree of human-land coordination [5,6,7]. In China, Shaanxi is rich in energy resources, its coal production ranks third in the country, and its oil and gas equivalent ranks first in the country. It is the country’s west-east coal, west-east gas transmission and west-east power transmission base, and it has a pivotal position in the protection of China’s energy supply security. The average GDP growth rate of Shaanxi Province from 1979 to 2019 was 10.3%. However, such high-speed growth was based on a large amount of energy consumption, and Shaanxi’s energy consumption structure was dominated by coal consumption. Environment and ecology have been significantly impacted by coal energy consumption. Shaanxi exhibits an extensive growth mode that will lead to the continuous deterioration of the province’s ecological environment vulnerability, which will not be conducive to its sustainable development and ecological civilization construction. At present, China is striving to achieve its “carbon peak” and “carbon neutrality” goals by 2030 and 2060, respectively, and realizing the energy consumption constraints and energy security constraints as well as the transformation toward green ecological development has become a problem that Shaanxi has to face. Due to the “carbon neutral” green energy wave, Shaanxi is moving from being a large energy province to being a strong energy province. To achieve the 2030 carbon peak and 2060 carbon neutrality targets, improving energy eco-efficiency is imperative.

In 1990, Schaltegger and Stum first proposed the concept of eco-efficiency [8], which is the ratio of economic growth to increased environmental impact. Subsequently, the World Business Council for Sustainable Development (WBSCD) [9] asserted that eco-efficiency aims to meet the needs of human society to survive and improve people’s quality of life by providing products or services with competitive price advantages, thereby reducing the impact of the process on resource utilization intensity and the ecological environment to the carrying capacity of the earth. Lin and Long [10] defined it as the efficiency of resource use and environmental pollution reduction in economic development. In general, the core idea of scholars in China and elsewhere regarding eco-efficiency is the combination of economy and resource environment, and its essence is to achieve as much economic output as possible through as little input of resources and the environment as possible [11,12]. In terms of implementing the new development concept of innovation, coordination, green, openness, and sharing, the research connotation of ecological efficiency should integrate multidimensional variables (energy economic growth, environmental impact, social welfare, and human well-being) that reflect an ecological civilization and people’s yearning for a better life into the ecological efficiency evaluation system. However, most existing measurement indicators omit the investigation of human well-being, which may overestimate the level of ecological efficiency as well. The measurement methods for energy eco-efficiency mainly include stochastic frontier analysis (SFA) [13,14,15,16] and data envelopment analysis (DEA), with DEA being the main method. In 1978, Charnes et al. [17] proposed the DEA model, which is also known as the Charnes–Cooper–Rhodes (CCR) model. The basic idea is to give a certain weight to nonhomogeneous inputs and then aggregate them. However, due to the limitations of traditional DEA models in dealing with pollutants and other undesirable outputs, scholars have proposed super-efficiency DEA models so that effective decision-making units (DMUs) can be further compared and organized [18]. They have also developed the slack-based measure (SBM) model [19,20], the super-SBM model [21,22] and the epsilon-based measure (EBM) model [23,24]. Furthermore, scholars have conducted further research on the index selection and processing methods for undesirable output, making the measurement results more accurate and more in line with production practice.

Scholars have conducted different levels of research on China’s eco-efficiency but mainly based on national, provincial, or larger unit levels (subregions and metropolitan areas) [25,26,27]. Additionally, there are few studies on the dynamic evolutionary characteristics and causes of urban eco-efficiency in smaller spatial units. A city is a complete basic spatial unit and the basic level of provincial development. Its behavior organization and operation have irreplaceable characteristics that cannot be replaced by provincial and larger geographical units, showing unique behavioral mechanisms and operational rules [28,29]. Due to the influence of economic development, city size, resource endowment and geographical location, the eco-efficiency in different regions shows a significant trend of spatial imbalance and polarization [30,31]. Under the new development pattern of domestic circulation as the main body, the cross-regional flow of labor, capital, energy, and other factors is more frequent, and the resulting ecological and environmental problems have broken through geographical boundaries [32], presenting a complex, multithreaded spatial association network structure [33,34]. The contradiction between ecological protection and people’s well-being is becoming increasingly obvious among regions. However, most existing studies use exploratory spatial data analysis (ESDA) and traditional spatial econometric analysis methods to analyze and examine the spatial distribution of eco-efficiency, considering only geographical proximity or the proximity effect [35,36]. It is difficult to have an overall grasp of the spatial correlations between cities’ eco-efficiency, which hinders the achievement of energy saving in Shaanxi. It is impossible to reveal the impact of the “relationship” of eco-efficiency on the spatial agglomeration of eco-efficiency, nor is it possible to further reveal the structural form and spatial clustering method of eco-efficiency. Therefore, this paper takes 10 prefecture-level cities in Shaanxi as the research object, integrates multidimensional variables of people’s livelihood and well-being that reflect an ecological civilization and people’s desire for a better life into the evaluation system for urban ecological efficiency, and quantitatively measures the ecological efficiency of urban well-being. On this basis, it dynamically identifies the spatial correlation network structure of well-being energy eco-efficiency (WEE) and its driving factors using social network analysis (SNA), which is of great theoretical and practical importance.

Here are the main innovations of this paper: First, multidimensional variables of people’s livelihood and well-being that reflect an ecological civilization and people’s desire for a better life are incorporated into the evaluation system for urban eco-efficiency to make the calculation results more accurate. Second, the authors select the most basic urban unit as a sample to study WEE in Shaanxi, compensating for the shortcomings of most previous studies that are based on provincial or larger geospatial units as research samples and that cannot reflect the development status, characteristics and regional differences of WEE more realistically and meticulously. Third, breaking through geographical proximity restrictions, we analyze the spatial structure, clustering methods and driving factors of WEE through SNA.

This paper is organized as follows. Section 2 discusses the materials and methods used in this study. Section 3 focuses on the empirical results. The conclusions and policy recommendations are discussed in Section 4.

## 2. Methods and Materials

### 2.1. DEA Model

This study uses a super-SBM model based on constant returns to scale to measure urban WEE. The SBM model is a nonradial and nonangular measurement method of the DEA model that fully considers the relaxation of input and output [37]. Additionally, the super-SBM model compensates for the shortcomings of the SBM model by distinguishing those DMUs with effective levels [38]. Here is the formula:

(1) $$\begin{array}{l} \min\rho =\frac{1-(\frac{1}{n})\sum_{i=1}^{n}\left(\frac{x^{-}}{x_{ik}}\right)}{1+\frac{1}{o_{1}+o_{2}}[\sum_{s=1}^{o_{1}}(\frac{y_{s}^{d}}{y_{sk}^{d}})+\sum_{p=1}^{o_{2}}\left(\frac{y_{p}^{v}}{y_{pk}^{v}})\right]}\\ s.t.\left\{ \begin{array}{l} x^{-}\geq \sum_{j=1,\neq k}^{m}\left(x_{ij}\lambda_{j}\right)\;i=1,\ldots ,n\\ y_{s}^{d}\leq \sum_{j=1,\neq k}^{m}\left(y_{ij}^{d}\lambda_{j}\right)\;s=1,\ldots ,o_{1}\\ y_{p}^{v}\geq \sum_{j=1,\neq k}^{m}\left(y_{pj}^{d}\lambda_{j}\right)\;p=1,\ldots ,o_{2}\\ \lambda_{j}>0 \end{array}\right. \end{array}.$$

Suppose that there are m DMUs, each DMU includes n inputs ($x_{ik},i=1,\ldots ,n$), $o_{1}$ expected outputs ($y_{sk}^{d},s=1,\ldots ,o_{1}$), and $o_{2}$ undesirable outputs ($y_{pk}^{v},p=1,\ldots ,o_{2}$), and $x^{-},y_{s}^{d},y_{p}^{v}$ are the slack values of inputs, expected outputs and undesirable outputs, respectively. ρ represents the ecological efficiency value of urban well-being. If ρ≥1, the evaluation unit is valid; if 0<ρ<1, the evaluation unit has an efficiency loss.

### 2.2. VAR Granger Test Model

Referring to the results of existing research [6], this paper chooses the vector autoregressive (VAR) Granger causality test method to discuss the relationship between the cities of Shaanxi and to provide the basis for the subsequent construction of the network. First, this paper defines the time series of the WEE of any two cities as $\left\{x_{t}\right\}$ and $\left\{y_{t}\right\}$ and then conducts a Granger causality test by establishing two VAR models. The models are as follows:

(2) $$x_{t}=c_{1}+\sum_{j=1}^{p}\alpha_{1j}x_{t-j}+\sum_{j=1}^{q}\beta_{1j}y_{t-j}+\varepsilon_{1t}$$

(3) $$y_{t}=c_{2}+\sum_{j=1}^{r}\alpha_{2j}x_{t-j}+\sum_{j=1}^{s}\beta_{2j}y_{t-j}+\varepsilon_{2t}$$

where, *c_i_, α_i_, β_i_ (i = 1, 2)* are the parameters to be estimated; *{*${ԑ}_{it}$*} (i = 1, 2)* is the standard normal distribution residual term; *p, q, r, s* is the lag order of autoregressive term. If the test results show that the city x is the Granger cause of city y, a directed connection is drawn between the two regions from region x to region y. Based on this method, the following will examine the pairwise relationship of the WEE of all cities in Shaanxi and derive the correlation matrix.

### 2.3. SNA Method

Social network refers to the relationship structure formed by resource dependence among many groups, organizations, and individuals in society. The SNA method is a technology used to analyze the relationship structure, nature, and attributes between network participants. The “relationship” between the participants is the basis of SNA, and the interaction and resource dependence between the participants produce collective behavior. SNA believes that actors and their behaviors are interdependent. The relationship between actors is a channel for resource transfer and flow. Under the background of urban agglomeration development characterized by division of labor and cooperation, urban agglomerations have evolved into urban networks. Based on the concept of the social network, the city is taken as a node. Using the “flow data” between nodes, the status and interaction of each node in the network are explained from the perspective of “relationship”. In contrast to traditional econometric methods, the SNA method is useful for studying the spatial correlation throughout a region [39]. In this paper, the actors in the correlation network of WEE are ten cities in Shaanxi Province, and the relationship is the calculation result of VAR. The main indicators and analysis methods are as follows:

(1) Overall network structure characteristic index

Network density is applied to explore the degree of closeness in the relationship of urban WEE between cities. Cities with higher values have closer relationships. Represented by *D*, the formula is as follows:

(4) $$D=\frac{L}{N\times (N-1)}$$

where the network consists of *L* actual relationships and *N* nodes.

Network efficiency is an indicator used to determine the redundancy of the network structure. The network with high network efficiency will have fewer redundant connections, resulting in better network stability. Represented by *E*, the formula is as follows:

(5) $$E=1-\frac{M}{\max(M)}$$

where *M* is the number of redundant correlations, and max (*M*) is its maximum.

Network hierarchy is applied to measure the degree of network perfection. The higher the value is, the more perfect the network, and the edge and subordinate areas play a greater role in the formation of the associated network. Represented by *H*, the formula is as follows:

(6) $$H=1-\frac{K}{\max(K)}$$

where *K* is the number of symmetries, and max (*K*) is its maximum.

(2) Individual network structure characteristic index

The characteristics of individual network structure describe the role and status of prefecture-level cities in the correlation network of WEE. Usually, degree centrality, betweenness centrality, and closeness centrality are applied to describe the characteristics of an individual network structure. Among them, degree centrality refers to how many points are directly connected to a focal point in the network, which can be categorized into the in-degree and out-degree in the directed graph. If a node has a high degree of centrality, it may be in the center of the whole network and have high control over and influence on the formation of the network and other nodes. Here is the calculation formula:

(7) $$D_{e}=\frac{n}{N-1}$$

where *D_e_* represents degree centrality, *n* is the number of points directly related to the region, and *N* is the maximum number of points directly connected to the region.

Betweenness centrality measures a node’s control over another in the transmission of elements such as information and resources. If a node is located in a shortcut from many other points to another point, the point has a high degree of control over the interconnection between other nodes. Therefore, the betweenness centrality is high, and the node may be located in the center of the network. Here is the calculation formula:

(8) $$C_{ABi}=\sum_{h=1}^{n}\sum_{j=1}^{n}\frac{g_{hj}\left(i\right)}{g_{hj}}$$

where *h ≠ j ≠ i* and *j < k*. *C_ABi_* represents betweenness centrality, *g_hj_* represents the number of shortcuts connecting node *h* and node *j*, and *g_hj_*(*i*) represents the number of shortcuts connecting node *h* and node *j* through point *i*.

Closeness centrality refers to a node’s independence from other nodes in the spatial association network. The greater the closeness centrality, the closer the node is to another node, and the more favorable it may be when transmitting the information. Here is the calculation formula:

(9) $$C_{AP}^{-1}=\sum_{j=1}^{n}d_{ij}$$

where *C_AP_*^−1^ represents closeness centrality and *d_ij_* is the shortcut distance from node *i* to node *j*.

(3) Block model

The block model in SNA is commonly used to examine the relationship by analyzing the size of each location. Suppose that we analyze the relationships among the members of location *B_k_*. When *B_k_* has *g_k_* agents, the total number of possible relationships within *B_k_* is *g_k_* (*g_k_* − 1). If there are *g* agents in the overall network, then the total number of relationships per *B_k_* location is *g_k_* (*g* − 1). Therefore, the expected relationship ratio of this location is (*g_k_* − 1)/(*g* − 1). On this basis, the location studied can be divided into four parts, as shown in Table 1.

(4) QAP model

In QAP, the basic data is in matrix form, and the correlation between matrices is determined by comparing the differences between the two matrices. Firstly, the correlation coefficient between two known matrices is calculated. Secondly, the rows and corresponding columns of one of the matrices are randomly permutated at the same time (not just permuting rows or columns, otherwise breaking the original data). Then the correlation coefficient between the permutation matrix and another matrix is calculated. this calculation process is repeated hundreds or even thousands of times, and a distribution of correlation coefficients will be obtained. Finally, the correlation coefficient observed in the first step is compared with the distribution of the correlation coefficient calculated by random rearrangement to see whether the observed correlation coefficient falls into the rejection area or the acceptance area. There is no strict assumption about the independence of independent variables in the QAP, which makes it more robust than parametric tests. Because the spatial correlation of WEE in Shaanxi shows a clear network structure, the proxy variables are also relational data. If ordinary linear regression is used to analyze the relationship between data, there will be multicollinearity problems, which will lead to parameter estimation errors. Therefore, to ensure the accuracy of the regression results, we use the QAP method to investigate the influencing factors of the spatial correlation network of WEE in Shaanxi. The specific model is set as follows:

(10) $$E(i,j)=F\left\{G(i,j),O(i,j),S(i,j),\left.U(i,j),L(i,j),D(i,j)\right\}\right.$$

where *E(i,j)* is the binary matrix transformed from the spatial correlation network, and *G(i,j)*, *O(i,j)*, S(i,j), *U(i,j)*, *L(i,j)*, *D(i,j)* represent the difference matrix of economic development level, degree of openness, industrial structure, urbanization, population and geographical adjacency, respectively.

### 2.4. Study Area

**Figure 1:**
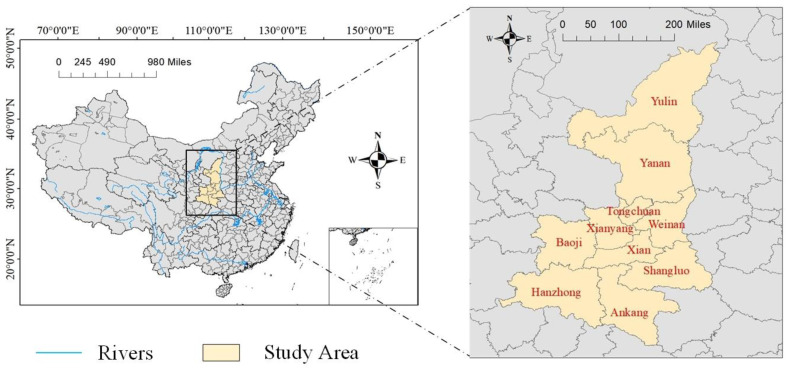
Distribution of 10 prefecture-level cities in Shaanxi Province.

Shaanxi Province is located in the northwest of China. It is rich in energy resources, and is an important base for China’s west-east coal transportation, west-east gas transmission and west-east power transmission. The energy consumption structure dominated by coal consumption in Shaanxi has led to the continuous deterioration of Shaanxi’s ecological environment vulnerability, which is not conducive to its sustainable development and ecological civilization construction. Therefore, this study selects 10 prefecture-level cities in Shaanxi as the research object, and explores the spatial network structure and driving factors of WEE. The distribution of ten cities in Shaanxi is shown in Figure 1.

### 2.5. Indicators and Data Sources

Strong sustainable development is a model of sustainable development of economic, social, and environmental resources. According to the demand for non-reducing development of environmental welfare in its theory, in the process of increasing economic growth and promoting the construction of ecological civilization, human beings should constantly transform and restore the environmental system and enhance the ability of environmental construction while damaging the ecological environment [40]. Therefore, this paper refers to the method proposed by Yang and Li [41] to measure the environmental construction index and environmental damage index, and incorporates them into the evaluation index system of WEE as input indicators and undesirable output respectively. Regional coordinated development is not only the sustainable development of the economy, resources, and environment, but also the well-being of people’s livelihoods. The ultimate goal of economic, resource and environmental development is to seek human welfare. Therefore, it is obviously impossible to truly measure the actual situation of China’s energy eco-efficiency by only selecting economic, resource, and environmental indicators to measure eco-efficiency [40]. Due to the strong subjectivity of the relevant indicators in the subjective well-being evaluation index and the large individual differences, this paper only constructs the livelihood well-being index system based on the objective well-being evaluation index. Based on the modernization theory, urbanization theory, government intervention theory and consumption theory, this paper refers to the research of Wang and Shi [42], Wang et al. [40] and Zhang et al. [43] and uses the entropy method to calculate the livelihood well-being index, as shown in Table 2.

Based on the above analysis and the principle of comprehensibility in science [44], the input indicators selected in this paper include four aspects: energy consumption, material capital, human capital and environmental construction index. The expected output includes the human well-being index and GDP. The undesirable output is the environmental damage index. The specific index system and selection method are shown in Table 3, and the descriptive statistics of WEE are shown in Table 4.

The change in the spatial network structure is a dynamic process that is affected by many factors, such as the differences in the regional economic development level, industrial structure, population and other factors. Based on relevant research and data availability, we select the variables of the economic development level, degree of openness, industrial structure, urbanization, population and geographical distance as the driving factors of the network. The descriptions of each variable are presented in Table 5. The research data come from the Shaanxi Statistical Yearbook, China Urban Construction Statistical Yearbook, and China City Statistical Yearbook as well as the statistical yearbooks of cities. Some missing data are supplemented through the interpolation method.

## 3. Results and Discussion

### 3.1. Measurement Results of WEE in Shaanxi

The Super-SBM model is used to calculate the WEE of 10 prefecture-level cities in Shaanxi from 2005 to 2019, as shown in Table 6. The WEE of Yan’an is the highest, with an average value of 0.992, which may be related to the strong support of the state and government for the ecological environment construction of Yan’an in recent years. The average values of Xi’an, Yulin and Tongchuan are all above 0.8, reaching a high level. Xi’an is the capital of Shaanxi and is in the central position. Yulin is the energy city of Shaanxi. Although the economic development level of Tongchuan is not as good as that of other cities, its environmental construction level is higher. The WEE of Ankang, Baoji, Hanzhong, Shangluo, Xianyang and Weinan is low, with an average value below 0.8. In terms of geographical location, the level of WEE in northern Shaanxi is generally high, and the cities rank in the top three. The cities with higher efficiency levels in the Guanzhong area are also cities with lower efficiency levels, and the polarization is more serious. Most cities in southern Shaanxi rank lower, and the efficiency level is low.

### 3.2. Spatial Correlation Network of WEE

**Figure 2:**
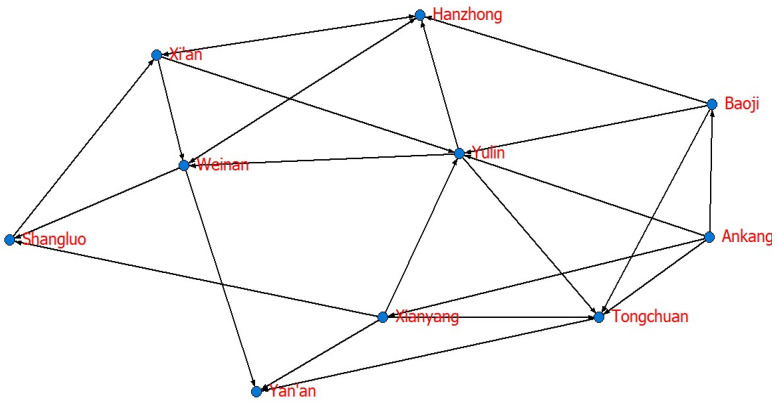
Spatial correlation network of WEE of 10 prefecture-level cities in Shaanxi Province.

Is the WEE of cities that are not geographically adjacent relevant? To answer this question, based on the VAR Granger causality test (significance level is 10%), this paper obtains the spatial correlation matrix, as shown in Table 7. Additionally, Figure 2 shows the spatial correlation network of WEE in Shaanxi from 2005 to 2019.

#### 3.2.1. Analysis of the Characteristics of the Overall Network Structure

The maximum number of relationships in 10 cities is 90 (10 × 9), while the correlation network has 24 actual relationships, and the network density is 0.2667. These results indicate that the tightness between cities is low, as shown in Figure 1. A better correlation and stability are needed for the entire network, and collaborative development can still be improved. The network correlation is 0.6, indicating that not all cities are included and its robustness needs to be improved. Having a network hierarchy of 0.7727 indicates that the entire network is highly hierarchical and that most cities are situated in edge regions, with only a few in the center. With a network efficiency of 0.6389, the network has several spare connections, and the spatial spillover of WEE is characterized by multiple superpositions. According to the above results, WEE’s spatial correlation network is not yet optimal, and there is still much room for improvement.

#### 3.2.2. Analysis of the Characteristics of the Individual Network Structure

**Figure 3:**
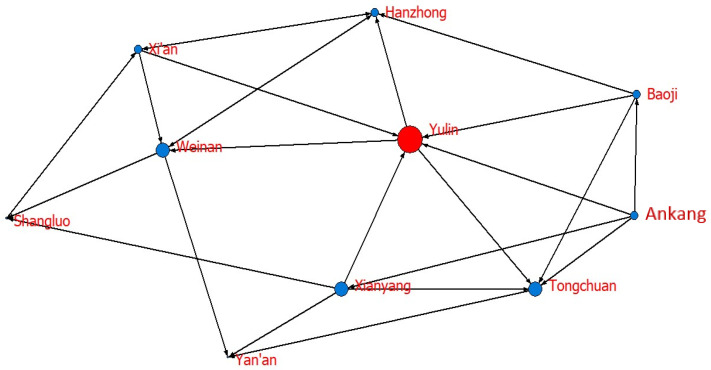
The network diagram of degree centrality.

**Figure 4:**
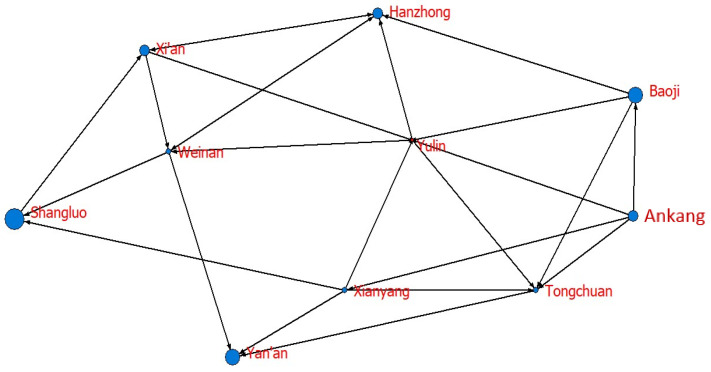
The network diagram of closeness centrality.

**Figure 5:**
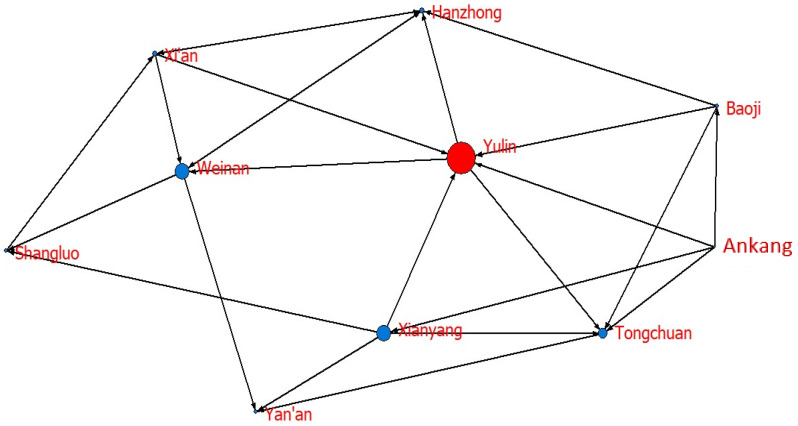
The network diagram of betweenness centrality.

To further explore the role of each city in the network, we calculate the degree centrality, closeness centrality, and betweenness centrality of each city, as shown in Table 8. At the same time, based on the calculation results and the Netdraw visualization tool, the network diagrams are drawn according to node size, as shown in Figure 3, Figure 4 and Figure 5.

Regarding degree centrality, the mean value of 10 cities is 48.888. In terms of specific cities, the top four cities are Yulin, Tongchuan, Xianyang, and Weinan, which all exceed the average value. Among them, Yulin has the highest degree centrality, 77.778. Yulin is at the center of the spatial correlation network and is bound up with other cities, as shown in Figure 3. It has high control over and influence on other nodes and the formation of networks. The degree centrality of Xi’an, Baoji, Hanzhong, and Ankang is relatively high, showing that these cities have a certain correlation with other cities; however, the connection is not strong. The degree centrality level of Yan’an and Shangluo is low, and they are marginalized in the whole network.

Regarding closeness centrality, the mean value of 10 cities in 65.862, ranging from 81.818 to 56.250, which is relatively balanced. Additionally, Figure 4 shows that except for Yulin, other cities have a high degree of closeness to the center, showing that the entire network is centered on Yulin and has a strong correlation. One possible reason is that Yulin is the main energy exporter of Shaanxi and has close contact with other cities, with Yulin and other cities having a strong influence on each other.

Regarding betweenness centrality, the mean value of 10 cities in Shaanxi is 6.667, with a maximum of 22.487 and a minimum of 1.323. Additionally, Figure 5 shows that Yulin has the highest betweenness centrality, and other cities have a smaller betweenness centrality. These results indicate that Yulin is the “bridge” of the network that generates correlations between other cities, while other cities are mostly subordinate. In general, cities with high economic development levels, high energy endowments and high environmental construction levels are also closely connected to each other. Cities with backward economic development, high energy consumption levels, and low environmental construction levels have weak correlations with other cities.

**Figure 6:**
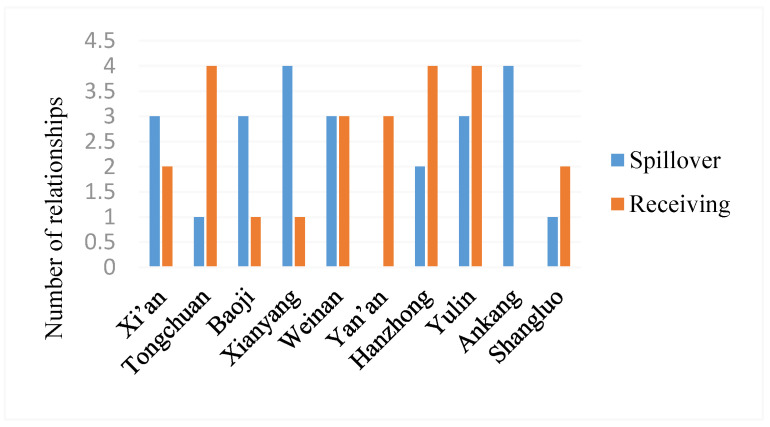
Spillover and receiving relationships of the spatial correlation network.

The spillover and receiving relationships of the network are illustrated in Figure 6, which shows that Tongchuan, Yan’ an, Hanzhong and Yulin benefit on the whole. They have fewer external spillovers and receive more spillovers from other cities, and Yan’an does not spill over outside but only unilaterally receives spillovers. These results indicate that the WEE of these cities is mainly affected by other cities. Xi’an, Baoji, Xianyang and Ankang are overall spillovers, and they have more spillovers to other cities and receive fewer spillovers from other cities, indicating that the impact of these cities on other cities is greater than the impact of other cities on these cities. In particular, the relationship between external spillovers and received spillovers in Weinan is the same. This result indicates that the influence of other regions on Weinan and its own influence on other regions are equal. Moreover, cities with higher WEE, such as Yan’an, Xi’an, Yulin and Tongchuan, do not have significant spillover effects, as expected. One possible reason is that they have the siphon effect and the polarization effect, absorbing more resources, talent, and technologies from other cities to develop themselves. Cities with low WEE, such as Xianyang and Ankang, can transport more resources and talent to other cities, having a strong external impact.

#### 3.2.3. Block Models

**Figure 7:**
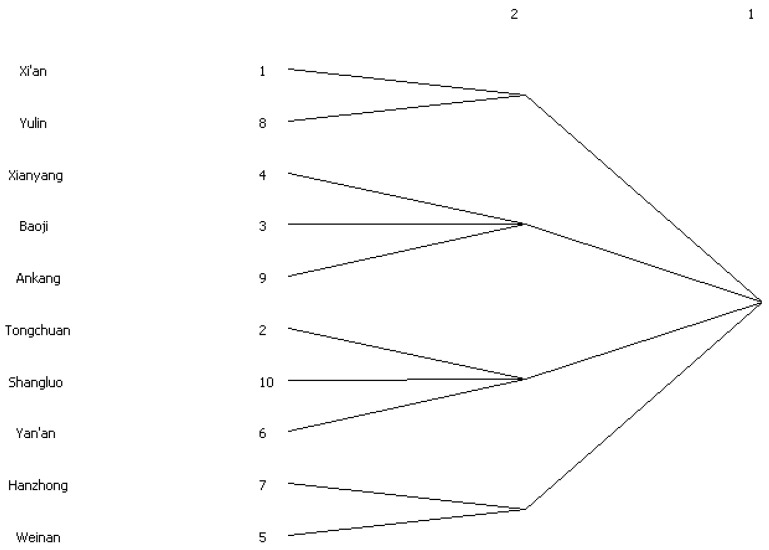
Clustering results of WEE of 10 prefecture-level cities in Shaanxi Province.

With 2 as the maximum cutting depth and 0.2 as the convergence standard, this paper divides the 10 cities into four plates based on their positions in the network. As presented in Figure 7, plate one consists of Xi’an and Yulin; plate two consists of Xianyang, Baoji and Ankang; plate three consists of Tongchuan, Shangluo and Yan’an; and plate four consists of Hanzhong and Weinan. The results show that the relationship between members in the network is not restricted by geographical divisions, which also confirms the feasibility of using SNA.

Table 9 shows the Characteristics of each plate. In this network, the 4 plates have 6 actual relationships, accounting for 14.3% of the total. There are 36 issued and received spillover relationships within the four plates, representing 85.7% of the total, indicating a significant spillover effect. Specifically, the total number of actual internal relationships within plate one is 1, the number of issued spillover relationships to other plates and the number of overflow relationships received from other plates are both 5, and there is a higher expected internal relationship ratio than there actually is, which is 11.11%. Plate one is a two-way overflow plate. The members of plate one have two-way spillover effects on intraplate members and other plate members and play a role as intermediaries in the network. The total number of actual internal relationships within plate two is 2. There are 9 issued spillover relationships, but no overflow relationship is received from outside plates, and there is a higher expected internal relationship ratio than there actually is, which is 22.22%. Plate two is a net overflow plate, which is dominated by spillover to other plates in the network. The total number of actual internal relationships within plate three is 1. There is 1 issued spillover relationship, there are 8 external overflow relationships received, and the expected internal relationship ratio is 22.22%, which is higher than the actual internal relationship ratio. Plate three is a main benefit plate, which mainly receives the spillover relationship of other plates. The total number of actual internal relationships within plate four is 2, there are 3 issued spillover relationships, there are 5 external overflow relationships received, and the expected internal relationship ratio is 11.11%, which is lower than the actual internal relationship ratio. Therefore, plate four is a broker plate, and the members of this plate are the link between the plates and play a “bridge” role in the network.

To further analyze the correlation between plates, this paper uses Ucinet to obtain the density matrix between the plates, and it uses the overall density value (0.2667) calculated in Section 3.2.1 as the threshold value. If the density between the plates is greater than 0.2667, the value is 1 and 0 otherwise. In Table 10, we obtain the image matrix.

As seen in Table 10, the WEE within plate one, plate two and plate four has a significant correlation; however, plate three does not have a significant correlation with the other three plates. Specifically, plate one mainly overflows into plate four; plate two mainly overflows into plate one and plate three; plate four mainly overflows into plate three; and plate three does not overflow to any other plates.

**Figure 8:**
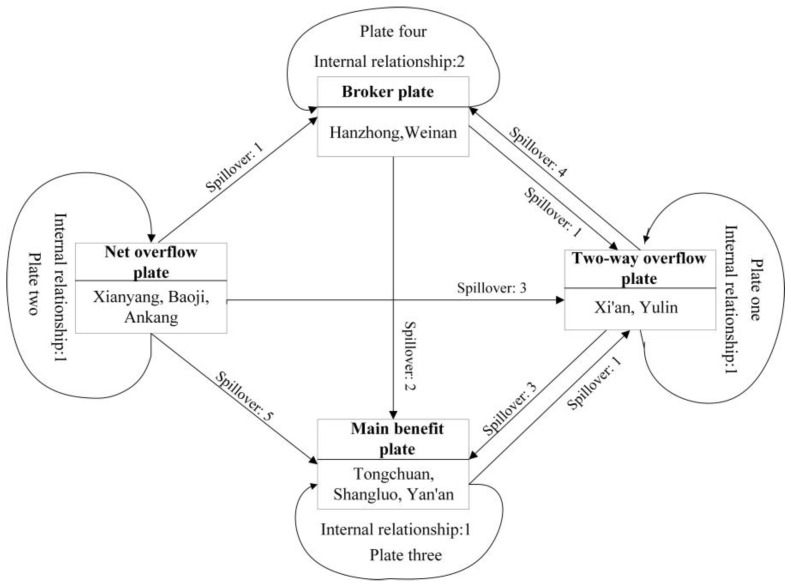
Correlations between the four plates.

Furthermore, we obtain the specific correlation between the four plates, as shown in Figure 8. Plate one and plate two have mainly outward spillover relationships, receiving less, and plate four and plate three mainly receive spillover from the remaining plates. In general, the net overflow plate includes Xianyang, Baoji and Ankang, and the two-way overflow plate includes Xi’an and Yulin, which play an “engine” role in the whole network. The broker plate, which includes Hanzhong and Weinan, plays an important “bridge” role. The two-way overflow plate receives spillovers from the net overflow plate and passes them on to the broker plate, which then passes them on to the main benefit plate, which includes Tongchuan, Shangluo and Yan’an. Moreover, all plate members have not been fully utilized, and the network as a whole still needs to be enhanced.

### 3.3. Driving Factors of the Spatial Correlation Network

#### 3.3.1. QAP Correlation Analysis

Table 11 shows the results of the QAP correlation analysis between the spatial correlation matrix of WEE and its influencing factors based on 5000 matrix random permutations. According to the calculation results, the correlation coefficients of economic development difference, industrial structure difference, and population difference are positive and significant at the 10%, 10%, and 5% levels, respectively. These results show that the greater the potential energy of economic flow and human capital flow between two places, the less similar the industrial structure, and the easier they are to be closely connected, thus exerting an important influence on the formation of the network. The correlation coefficients of urbanization difference and geographical adjacency are also positive, which indicates that the greater the urbanization difference between two places and the closer the geographical proximity, the more likely they are to be associated. This speculation has been initially confirmed, but the significance is not strong. Surprisingly, the coefficient of openness difference is negative, indicating that a stronger degree of openness has a certain inhibitory effect on the formation of the network, which is inconsistent with the initial conjecture. However, the significance of the result is not strong; thus, further investigation is needed. This paper further conducts QAP correlation analysis on each driving factor matrix. The results, which are presented in Table 12, show that some driving factor matrices are also correlated with each other. To avoid the coefficient bias caused by multicollinearity, it is necessary to further conduct QAP regression analysis on the driving factors.

#### 3.3.2. QAP Regression Analysis

Based on the correlation analysis, multivariate QAP regression analysis is applied to perform stepwise regression analysis on the dependent variable matrix and the independent variable matrix. As presented in Table 13, the results show that the adjusted determination coefficient is 0.058 after 5000 random replacements, indicating that the explanatory power of each driving factor with respect to the spatial correlation network reaches 5.8%.

Table 13 shows that the regression coefficient between the industrial structure difference and the correlation network of WEE is the highest, followed by the population size difference and the economic development difference. The coefficient is positive and significant at the 10% level, and it is the main driving factor affecting the network. The regression results of geographical adjacency are consistent with the results of the correlation analysis, and the significance is still not strong, which verifies that the correlation network of WEE has broken through the conjecture of the geographical proximity effect, indicating that the spatial network structure is not at the primary stage but at a higher level. In addition, the coefficient of the degree of openness is significant at the 5% level, indicating that the higher the openness degree, the worse of the construction of the spatial network. One reason may be that cities with a higher degree of openness are more likely to attract foreign investment and cause the transfer of low-end industries, resulting in consuming more energy, discharging more environmental pollution, impeding technological progress and energy inefficiency, thereby reducing WEE and inhibiting the formation of the network.

## 4. Conclusions and Policy Implications

### 4.1. Conclusions

We use the super-SBM model to measure the WEE of 10 cities in Shaanxi, China. Then, based on the VAR Granger causality test and SNA, this paper depicts the characteristics of the spatial correlation network structure and spatiotemporal evolutionary trend of WEE. Additionally, it uses the QAP method to identify the driving factors affecting the spatial correlation network structure. The main conclusions are as follows:

First, the overall level of WEE in Shaanxi is low, and the interregional development difference is large. The WEE in northern Shaanxi is the highest, followed by Guanzhong and southern Shaanxi. This situation poses new challenges for improving WEE.

Second, WEE has broken through the geographical proximity effect and gradually developed from “point” to “line” and “mesh”, showing a complex, multithreaded spatial correlation network structure. However, the closeness between cities is low, and a more stable and correlated network is needed. Yulin is at the center of the entire network and plays the role of “leader”. The centrality level of Yan’an and Shangluo is low, and they are marginalized in the whole network. Therefore, we should strengthen the cooperation between cities, maximize the spillover effect of “leading” cities, overcome the limitation of geographical distance, promote the formation of the network, and jointly improve WEE.

Third, Xi’an and Yulin belong to the two-way overflow plate, playing the role of middleman. Xianyang, Baoji, and Ankang comprise the net overflow plate, mainly spilling over to other plates. Tongchuan, Shangluo and Yan’an belong to the main benefit plate and mainly receive the spillover relationship of other plates. Hanzhong and Weinan comprise the broker plate, which acts as a “bridge” in the whole network. Therefore, the characteristics of the plates in the network should be fully understood, in order to formulate differentiated paths for improving the WEE.

Fourth, through QAP correlation and regression analysis, we find that the differences in the economic development, the industrial structure, population, and the degree of openness are the main driving factors of the network. The greater the potential energy of economic flow and human capital flow between two places is, the less similar the industrial structure, and the easier it is to have close contact, which has an important impact on the formation of the network. At the same time, the spatial correlation network of WEE has broken through the geographical proximity effect and is no longer at the primary stage but at a higher level.

### 4.2. Policy Implications

First, the government should give full play to its guidance and supervision functions, increase the protection of regional environmental construction, and improve the level of regional WEE. The government should implement relevant preferential policies based on the specific conditions of each region, issue relevant subsidies and preferential policies for the relatively backward economic development in southern Shaanxi, and actively guide local enterprises to carry out technological innovation under the constraints of the original low energy consumption and low pollution, develop clean energy and improve the economic development level. For the northern Shaanxi and Guanzhong regions with higher economic development levels and a lower WEE, the government should strengthen environmental supervision, improve energy efficiency, and enhance environmental protection and construction level.

Second, the government should accurately locate the position of each city in the associated network, fully tap its advantages, and actively carry out mutually beneficial cooperation between cities to improve the network correlation, enhance network density, and optimize the overall network structure. Plate one (two-way overflow plate) should give full play to its comparative advantage, effectively play the role of “leader”, and actively undertake the task of driving the development of other sectors. Yulin is the largest energy city in Shaanxi, and in recent years, it has had the fastest GDP growth. As the center of the spatial correlation network, Yulin should give full play to its control and leading role, strengthen its assistance to and cooperation with marginal cities (mainly Yan’an and southern Shaanxi), promote its economic development and human well-being, and then improve the correlation level and robustness of the whole network. As the capital city of Shaanxi, Xi’an has strong economic, technological, and talent advantages, but its betweenness centrality is only 3.108, ranking sixth in Shaanxi. Therefore, it should fully tap its resources and political advantages, actively carry out mutually beneficial cooperation, and promote the construction of central cities, thus driving the development of surrounding cities. Plate two (net overflow plate), in addition to acting as the “power source” of the entire network, should be accumulated to strengthen its own construction, increase talent attraction, promote technological progress and economic development through the introduction of the advanced experience of other sectors, and form a late-mover advantage. Plate three (main benefit plate) should make full use of the spillover effect of other plates on it, strive to strengthen its own construction, and transmit energy for its plate. Plate four (broker plate) should give full play to its own unique geographical advantages, enhance the level of openness, and actively communicate with other plates.

## Figures and Tables

**Table 1:** Plate classification.

The Relationship Ratio within the Position	Percentage of Relationships Accepted by the Location	
	≈0	>0
≥(*g_k_* − 1)/(*g* − 1)	Two-way overflow plate	Main benefit plate
<(*g_k_* − 1)/(*g* − 1)	Net overflow plate	Broker plate

**Table 2:** Evaluation indexes for human well-being.

Objective Level	First Grade Indexes	Second Grade Indexes	Third Grade Indexes
Human well-being index	Economic well-being	Economic income	Local fiscal revenue (CNY million), per capita disposable income of urban residents (CNY), per capita disposable income of rural residents (CNY)
	Cultural and educational well-being	Cultural activities	Every 10,000 people have the number of library collections (copy), the number of mass art museums, the number of cultural centers, the number of cultural stations(unit), the radio population coverage rate, and the TV population coverage rate (%).
		Education level	Per capita years of education (years)
	Social development well-being	Infrastructural construction	Highway mileage (km), the number of fixed telephone users (families), the number of mobile phone users (families), number of Internet broadband access users (families), number of buses per 10,000 people (person), number of hospital beds (bed)
		City development	local fiscal expenditure(CNY million), population density(person/km^2^), urbanization rate(%), number of employed persons (persons)

**Table 3:** Evaluation index system of WEE.

Index	Variables	Indicator Explanation
Input	Energy consumption	Product of energy consumption per unit GDP and GDP (Ten thousand tons of standard coal)
	Material capital	Calculated by using the perpetual inventory method and fixed asset investment (CNY billion)
	Human capital	It is expressed as the product of the number of employed people at the end of the year and the years of education per capita.
	Environmental construction index	11 indicators including the green coverage rate of the urban built-up area (%), per capita green area (m^2^), control area of soil and water loss in the year (10^3^ m^2^), investment in water conservancy construction (10^4^ CNY), afforestation area of barren hills and sands (hectare), standard treatment capacity of industrial wastewater (ton), removal capacity of industrial smoke and dust (ton), comprehensive utilization rate of industrial solid waste (%), removal capacity of industrial sulfur dioxide (ton), total amount of sewage treatment (10^4^ m^3^), and harmless treatment capacity of domestic waste (million tons)
Expected output	Human well-being index	The human well-being index is constructed from three dimensions: economic well-being, cultural and educational well-being and social development well-being, as shown in Table 2.
	GDP	GDP at constant prices based on 2005 (CNY billion)
Undesirable output	Environmental damage index	7 indicators including the PM_2.5_ concentration (µg/m^3^), CO_2_ emissions (million tons), SO_2_ emissions (ton), industrial wastewater emission (million tons), industrial smoke and dust emissions (ton), industrial solid waste production (million tons) and chemical fertilizer application (ton)

**Table 4:** Descriptive statistics of WEE.

Index	Variables	Sample Size	Unit	Mean	SD	Max	Min
Input	Energy consumption	150	Ten thousand tons of standard coal	1487.641	1376.191	6028.793	117.731
	Material capital	150	CNY billion	6009.112	7243.440	39,709.285	344.900
	Human capital	150	-	658.717	840.316	4462.105	94.386
	Environmental construction index	150	-	0.173	0.102	0.527	0.039
Expected output	human well-being index	150	-	915.030	983.865	5629.000	68.080
	GDP	150	CNY billion	0.192	0.069	0.537	0.089
Undesirable output	Environmental damage index	150	-	0.185	0.144	0.609	0.019

**Table 5:** Variable matrix specification.

Variables	Sign	Specification
Economic development differences	*G(i,j)*	Calculate the difference between the average GDP of two cities in Shaanxi from 2005 to 2019, and construct the difference matrix with the row mean as the threshold.
Openness differences	*O(i,j)*	Calculate the average value of the actual utilization of foreign capital in each city in Shaanxi from 2005 to 2019, and use the median as the dividing point to divide cities into those with high and low openness. The same high openness city is set to 1 and 0 otherwise. On this basis, the urban openness network is built.
Industrial structure differences	*S(i,j)*	Calculate the difference between the mean value of the ratio of the secondary industry to the regional GDP of two cities in Shaanxi from 2005 to 2019, and construct the difference matrix with the row mean as the threshold.
Urbanization differences	*U(i,j)*	Calculate the difference between the proportion of the urban population in the total population of two cities in Shaanxi from 2005 to 2019, and construct the difference matrix with the row mean as the threshold.
Population differences	*L(i,j)*	Calculate the difference between the total population of two prefecture-level cities in Shaanxi from 2005 to 2019, and construct the difference matrix with the row mean as the threshold.
Geographical adjacency	*D(i,j)*	Based on Baidu Map, geographically adjacent cities are set to 1 and otherwise 0, to obtain a geographical distance network.

**Table 6:** The WEE values of 10 cities in Shaanxi from 2005 to 2019.

Year	Xi’an	Tongchuan	Baoji	Xianyang	Weinan	Yan’an	Hanzhong	Yulin	Ankang	Shangluo	Mean
2005	0.811	1.022	0.684	0.664	1.028	0.940	0.694	1.004	0.749	1.161	0.8757
2006	1.072	1.005	0.716	0.701	0.978	0.942	0.838	0.848	0.742	1.007	0.8849
2007	1.015	1.040	0.753	0.703	1.012	1.016	1.009	1.106	0.776	0.858	0.9288
2008	0.808	1.007	0.773	0.674	0.922	1.006	1.007	1.018	0.764	0.813	0.8792
2009	0.831	0.763	0.744	0.690	1.000	1.019	1.001	0.876	0.772	0.723	0.8419
2010	0.820	1.003	0.724	0.649	0.592	1.015	0.972	0.854	0.739	0.743	0.8111
2011	0.870	0.991	0.715	0.672	0.540	0.969	0.856	0.821	0.753	0.760	0.7947
2012	0.842	0.670	0.753	0.678	0.548	1.077	0.752	0.819	0.757	0.731	0.7627
2013	0.879	0.666	0.679	0.655	0.523	0.935	0.700	0.808	0.734	0.743	0.7322
2014	0.879	0.665	0.725	0.669	0.542	0.923	0.690	0.810	0.734	0.671	0.7308
2015	0.906	0.695	0.770	0.683	0.552	1.001	0.653	0.887	0.751	0.703	0.7601
2016	0.966	0.686	0.838	0.747	0.566	1.020	0.648	0.911	0.792	0.644	0.7818
2017	1.036	0.771	0.979	0.961	0.546	1.010	0.637	0.963	1.019	0.714	0.8636
2018	0.958	1.004	0.981	1.063	0.578	1.026	0.632	1.043	0.740	0.677	0.8702
2019	0.981	0.790	1.030	1.002	0.639	0.977	0.657	0.782	1.074	0.785	0.8717
Mean	0.912	0.852	0.791	0.747	0.704	0.992	0.783	0.903	0.793	0.782	0.8259
Rank	2	4	6	9	10	1	7	3	5	8	-

**Table 7:** Spatial correlation matrix of WEE.

	Xi’an	Tongchuan	Baoji	Xianyang	Weinan	Yan’an	Hanzhong	Yulin	Ankang	Shangluo
Xi’an	1	0	0	0	1	0	1	1	0	0
Tongchuan	0	1	0	0	0	1	0	0	0	0
Baoji	0	1	1	0	0	0	1	1	0	0
Xianyang	0	1	0	1	0	1	0	1	0	1
Weinan	0	0	0	0	1	1	1	0	0	1
Yan’an	0	0	0	0	0	1	0	0	0	0
Hanzhong	1	0	0	0	1	0	1	0	0	0
Yulin	0	1	0	0	1	0	1	1	0	0
Ankang	0	1	1	1	0	0	0	1	1	0
Shangluo	1	0	0	0	0	0	0	0	0	1

**Table 8:** Centrality analysis of spatial correlation networks.

City	Degree Centrality				Closeness Centrality		Betweenness Centrality	
	Out-Degree	In-Degree	Centrality	Rank	Centrality	Rank	Centrality	Rank
Xi’an	3	2	44.444	3	64.286	3	3.108	6
Tongchuan	1	4	55.556	2	69.231	2	6.415	4
Baoji	3	1	44.444	3	60.000	4	2.778	7
Xianyang	4	1	55.556	2	69.231	2	11.376	2
Weinan	3	3	55.556	2	69.231	2	10.979	3
Yan’an	0	3	33.333	4	60.000	4	2.315	8
Hanzhong	2	4	44.444	3	64.286	3	3.571	5
Yulin	3	4	77.778	1	81.818	1	22.487	1
Ankang	4	0	44.444	3	64.286	3	1.323	9
Shangluo	1	2	33.333	4	56.250	5	2.315	8
Mean	2.4	2.4	48.888	-	65.862	-	6.667	-

**Table 9:** Characteristics of each plate.

Plate	City	Number of Actual Relationships within Plates	Spillover	Reception	Expected Internal Relationship Ratio/%	Actual Internal Relationship Ratio/%	Characteristic
Plate one	Xi’ an, Yulin	1	5	5	11.111	9.090	Two-way overflow plate
Plate two	Xianyang, Baoji, Ankang	2	9	0	22.222	18.182	Net overflow plate
Plate three	Tongchuan, Shangluo, Yan’ an	1	1	8	22.222	10.000	Main benefit plate
Plate four	Hanzhong, Weinan	2	3	5	11.111	20.000	Broker plate

**Table 10:** Density matrix and image matrix between plates.

Plate	Density Matrix				Image Matrix			
	Plate One	Plate Two	Plate Three	Plate Four	Plate One	Plate Two	Plate Three	Plate Four
Plate one	0.500	0.000	0.167	1.000	1	0	0	1
Plate two	0.500	0.333	0.556	0.167	1	1	1	0
Plate three	0.167	0.000	0.167	0.000	0	0	0	0
Plate four	0.250	0.000	0.333	1.000	0	0	1	1

**Table 11:** QAP correlation analysis results of the spatial correlation matrix *E(i,j)* and its driving factors.

Variables	Correlation Coefficient	Significance	SD	Min	Max
*G(i,j)*	0.189	0.053	0.101	0.344	−0.326
*O(i,j)*	−0.081	0.287	0.089	0.282	−0.201
*S(i,j)*	0.174	0.075	0.108	0.325	−0.379
*U(i,j)*	0.023	0.517	0.103	0.325	−0.379
*L(i,j)*	0.191	0.044	0.102	0.342	−0.363
*D(i,j)*	0.034	0.461	0.099	0.395	−0.326

**Table 12:** QAP correlation analysis results of the driving factors.

	*G* *(i,j)*	*O* *(i,j)*	*S* *(i,j)*	*U* *(i,j)*	*L* *(i,j)*	*D* *(i,j)*
*G(i,j)*	1					
*O(i,j)*	0.177 (0.209)	1				
*S(i,j)*	0.170 (0.152)	−0.024 (0.406)	1			
*U(i,j)*	0.033 (0.437)	0.083 (0.417)	0.377 ** (0.011)	1		
*L(i,j)*	0.213 (0.137)	0.143 (0.286)	0.048 (0.391)	−0.042 (0.475)	1	
*D(i,j)*	0.112 (0.239)	0.122 (0.289)	−0.104 (0.260)	−0.286 ** (0.040)	0.168 (0.127)	1

Note: The numbers in parentheses indicate *p* values, ** Significant at 5% levels, respectively.

**Table 13:** QAP regression analysis results of the spatial correlation matrix *E(i,j)* and its driving factors.

Variable	Non-Standardized Regression Coefficients	Standardized Regression Coefficient	Significance Probability Value	P_1_	P_2_
*G(i,j)*	0.138373	0.152542	0.074	0.074	0.927
*O(i,j)*	−0.145850	−0.155476	0.048	0.952	0.048
*S(i,j)*	0.149633	0.169018	0.073	0.073	0.928
*U(i,j)*	−0.024785	−0.027996	0.388	0.612	0.388
*L(i,j)*	0.138698	0.156473	0.054	0.054	0.946
*D(i,j)*	0.002871	0.003165	0.462	0.426	0.539

## Data Availability

The datasets of this paper are available from the corresponding author on reasonable request.
